# External Validation of a Nomogram That Predicts the Pathological Diagnosis of Thyroid Nodules in a Chinese Population

**DOI:** 10.1371/journal.pone.0065162

**Published:** 2013-06-04

**Authors:** Ridong Wu, Liling Zhu, Wen Li, Qing Tang, Fushun Pan, Weibin Wu, Jie Liu, Chen Yao, Shenming Wang

**Affiliations:** 1 Department of Vascular and Thyroid Surgery, The First Affiliated Hospital of Sun Yat-sen University, Guangzhou, P. R. China; 2 Department of Breast Surgery, Breast Tumor Center, Sun Yat-sen Memorial Hospital, Sun Yat-sen University, Guangzhou, P. R. China; 3 Department of General Surgery Laboratory, The First Affiliated Hospital of Sun Yat-sen University, Guangzhou, P. R. China; 4 Department of Plastic and Reconstructive Surgery, The First Affiliated Hospital of Sun Yat-sen University, Guangzhou, P. R. China; 5 Department of Medical Ultrasonics, The First Affiliated Hospital of Sun Yat-sen University, Guangzhou, P. R. China; Consiglio Nazionale delle Ricerche (CNR), Italy

## Abstract

**Introduction:**

Nomograms are statistical predictive models that can provide the probability of a clinical event. Nomograms have better performance for the estimation of individual risks because of their increased accuracy and objectivity relative to physicians’ personal experiences. Recently, a nomogram for predicting the likelihood that a thyroid nodule is malignant was introduced by Nixon. The aim of this study was to determine whether Nixon’s nomogram can be validated in a Chinese population.

**Materials and Methods:**

All consecutive patients with thyroid nodules who underwent surgery between January and June 2012 in our hospital were enrolled to validate Nixon’s nomogram. Univariate and multivariate analyses were used to identify the risk factors for thyroid carcinoma. Discrimination and calibration were employed to evaluate the performance of Nixon’s model in our population.

**Results:**

A total of 348 consecutive patients with 409 thyroid nodules were enrolled. Thyroid ultrasonographic characteristics, including shape, echo texture, calcification, margins, vascularity and number (solitary vs. multiple nodules), were associated with malignance in the multivariate analysis. The discrimination of all nodules group, the group with a low risk of malignancy (predictive proportion <50%) and the group with a high risk of malignancy (predictive proportion ≥50%) using Nixon’s nomogram was satisfactory, and the area under the receiver operating characteristic curve of the three groups were 0.87, 0.75 and 0.72, respectively. However, the calibration was significant (p = 0.55) only in the high-risk group.

**Conclusion:**

Nixon’s nomogram is a valuable predictive model for the Chinese population and has been externally validated. It has good performance for patients with a high risk of malignancy and may be more suitable for use with these patients in China.

## Introduction

The thyroid nodule is a quite common clinical problem, and its incidence is rapidly increasing in many areas of the world [1]. A population study suggested that 4–7% of the United States adult population has a palpable thyroid nodule [2], and the incidence of thyroid nodules detected by ultrasonography in some regions of China has been shown to range from 29.14% to 37.24% [3], [4], [5]. Only 5% of these nodules are malignant [6], but the malignancy rate may be increased if the patient has other specific characteristics, such as a family history of carcinoma, radiation exposure and specific ultrasound features. Therefore, the accurate diagnosis and the rational management of thyroid nodules are very important.

The family history, physical and laboratory examinations, thyroid ultrasonography and a fine needle aspiration biopsy (FNAB) comprise the standard evaluation for nodular thyroid disease [7]. FNAB is the most accurate and cost-effective method for evaluating thyroid nodules [8], but the results may vary greatly depending on the skill of the physician who takes the biopsy and the skill of the cytologist. Nomograms are statistical predictive models that can be used to calculate the probability of a clinical event [9]. Currently, greater numbers of physicians are beginning to use nomograms to assess individual risks because of their increased accuracy and objectivity relative to doctors’ personal experiences and the current staging system [10], [11]. Some nomograms can be used to assess nodular thyroid disease, including the nomograms of Stojadinovic [7], Banks [12], Nixon [13], [14] and Tomei [15]. Nixon’s nomogram, developed in 2012, is based on age, thyroid-stimulating hormone (TSH) level, size (based on ultrasonography), shape, echo texture, calcification, margin and vascularity. This nomogram performed well in an internal validation study. Nevertheless, external validation is crucial to ensure applicability to patients from different populations [16]. Therefore, Nixon’s nomogram must be extensively validated to assess its usefulness.

In this study, we were the first to validate Nixon’s nomogram in Chinese patients with thyroid nodules who underwent surgery and had a pathological diagnosis. Our aim was to determine whether this nomogram can be validated for use with the Chinese population and whether this tool can help predict the malignancy of nodular thyroid disease.

## Materials and Methods

### Study Population

All consecutive patients with thyroid nodules who underwent surgery and had a pathological diagnosis between January and June 2012 in the First Affiliated Hospital of Sun Yat-sen University were identified. Patients were excluded if they met any of the following criteria: (i) the thyroid ultrasound images or TSH values were missing from the clinical records or (ii) the laboratory examinations, ultrasound images or surgery were not completed at our hospital. A total of 348 patients with 409 thyroid nodules were finally included. Clinical and ultrasonographic characteristics, such as age, gender, TSH values, pathological diagnosis, tumor size, number (solitary vs. multiple nodules), shape, echo texture, calcification, margins and vascularity, were collected.

### Statistical Analysis

Univariate and multivariate analyses were performed to determine the risk of malignant thyroid nodules. Student’s t-test was used for continuous variables, such as age, TSH and tumor size, and the chi-square test or Fisher’s exact test was employed for categorical variables, such as gender, pathologic diagnosis, number (solitary vs. multiple nodules), shape, echo texture, calcification, vascularity and margins. All indexes were included in the multivariate analysis. All tests were two sided, and p-values <0.05 were considered significant.

Nixon’s nomogram was obtained from his article [14]. An individual’s probability of thyroid cancer can be calculated as follows: locate the patient’s variables on the corresponding lines and then draw vertical lines from one index up to the points line. Add up the points for all variables, find the total point value on the total points line, and then draw a vertical line from the total points line to the probability of thyroid cancer line and identify the final probability. The performance of Nixon’s nomogram for our study population was quantified based on discrimination and calibration. Discrimination was used to determine whether the individual predictions were correct. The level of discrimination was estimated using the area under the receiver operating characteristic (ROC) curve (AUC). Calibration was used to determine whether the observed frequencies were concordant with the predicted probabilities. The level of calibration was estimated using a calibration curve constructed by plotting the probability predicted by the nomogram against the actual frequency. A p-value >0.05 was considered well calibrated, meaning that there was no significant difference between the previous two items. We also calculated the average errors (E-aver) and maximal errors (E-max) for the two groups.

Univariate and multivariate statistical analyses and ROC curve calculations were performed in SPSS 18.0 (SPSS, Chicago, IL, USA), and calibration was performed in R (http://cran.r-project.org).

### Ethical Considerations

Patients provided written informed consent prior to being registered in this study. This retrospective study was approved by the institutional review board and the ethical committee of the First Affiliated Hospital of Sun Yat-sen University (Guangzhou, China), and no ethical objections were raised.

## Results

A total of 348 consecutive patients with 409 thyroid nodules from the First Affiliated Hospital of Sun Yat-sen University were enrolled in this study. The clinical and pathological characteristics and the parameters from the thyroid ultrasound images for the validation cohort and Nixon’s nomogram cohort are shown in Table 1. In the validation cohort, 69.4% of the nodules were benign, and 30.6% were malignant. The median age of patients was 46 years (range, 12–96 years), and the median tumor size was 2 cm (range, 0.2–9 cm). Compared with the percentage in Nixon’s cohort, we observed a significantly higher percentage of benign thyroid nodules. In addition, number (solitary vs. multiple nodules), shape, echo texture, calcification and vascularity were also different between the two cohorts.

**Table 1:** Characteristics of validation cohort and Nixon’s nomogram cohort.

	Validation cohort	Nixon cohort^&^	P value*
thyroid nodules	409(100%)	182(100%)	
Age(years)^$^			
Median	46	56	NA
Range	12–96	16–89	
Gender^$^			
Male	95(27.3%)	52(32.9%)	0.198
Female	253(72.7%)	106(67.1%)	
TSH(mIU/ml)			
Median	1.31	1.52	NA
Range	0.007–21.26	0.05–24.8	
Pathologic diagnosis			
Benign	284(69.4%)	45(28.4%)	<0.001
malignant	125(30.6%)	113(71.6%)	
Tumor size(cm)			
Median	2	1.8	NA
Range	0.2–9	0.5–6.5	
Solitary			
Yes	225(55.0%)	36(19.8%)	<0.001
No	184(45.0%)	146(80.2%)	
Shape			
Oval	355(87.1%)	132(72.5%)	<0.001
Taller than wide	34(7.9%)	15(8.2%)	
Variable	20(5%)	35(19.3%)	
Echo texture			
Hypoechoic	193(47.2%)	79(43.4%)	<0.001
Isoechoic	23(5.6%)	69(37.9%)	
Mixed	193(47.2%)	34(18.7%)	
Calcification			
None	261(63.8%)	92(50.5%)	<0.001
Microscopic	80(19.6%)	72(39.6%)	
Coarse	68(16.6%)	18(9.9%)	
Margins			
Well defined	286(69.9%)	115(63.2%)	0.105
Poorly defined	123(30.1%)	67(36.8%)	
Vascularity			
Hypervascular	152(37.2%)	32(17.6%)	<0.001
Hypovascular	236(57.7%)	52(28.6%)	
others	21(5.1%)	72(53.8%)	

NA: non available; TSH: thyroid-stimulating hormone.

$ these items are based on individual patient data.

&These data were cited from Nixon’s article [11].

* P values were obtained using the chi-square test.

Univariate analyses revealed that the TSH levels, tumor size, number (solitary vs. multiple nodules), shape, echo texture, calcification, margins and vascularity were strongly associated with malignancy, but there were no differences in age or gender. All significant risk factors were included in the multivariate analysis. A binary logistic regression analysis revealed that number (solitary vs. multiple nodules), shape, calcification, echo texture, margin and vascularity were associated with malignancy (Table 2).

**Table 2:** Univariate and multivariate analysis comparing benign thyroid nodules to malignant thyroid nodules in patients*.

	Univariate analysis			Multivariate analysis	
	Benign thyroidNodules, n = 284	Malignant thyroidNodules, n = 125	P	OR	P
Patient age(years)$			0.187		NS
Median(range)	48(12–96)	43(18–77)			
Standard deviation	13.57	12.20			
Gender$					
Male	65	30	0.938		NS
Female	172	81			
TSH(mIU/ml)					
Median(range)	1.10(0.007–21.26)	1.58(0.044–15.76)	0.025		NS
Standard deviation	1.52	1.96			
Tumor size(cm)					
Median(range)	2.4(0.2–9)	1.3(0.3–8.9)	0.030		NS
Standard deviation	1.58	1.25			
Solitary					
No	159	25	<0.001	Reference	
Yes	125	100		8.258	<0.001
Shape					
Oval	273	82	<0.001	Reference	
Variable	5	15		61.152	<0.001
Taller than wide	6	28		97.158	<0.001
Echo texture					
Isoechoic	18	5	<0.001	Reference	
Mixed	161	32		8.964	<0.001
Hypoechoic	105	88		7.642	<0.001
Calcification					
None	226	35	<0.001	Reference	
Coarse	48	20		3.968	0.003
Microscopic	10	70		42.954	<0.001
Margins					
Well defined	227	59	<0.001	Reference	
Poorly defined	57	66		3.255	0.005
Vascularity					
Hypovascular	187	49	<0.001	Reference	
Hypervascular	79	73		0.405	0.030
Others	18	3		0.057	0.017

OR: odds ratio; NS: non significant; TSH: thyroid-stimulating hormone.

* Student’s T-tests used for continuous variables and Chi-squared test for categorical variables. All statistical tests were two-sided.

$ these items are based on individual patient data.

**Figure 1:**
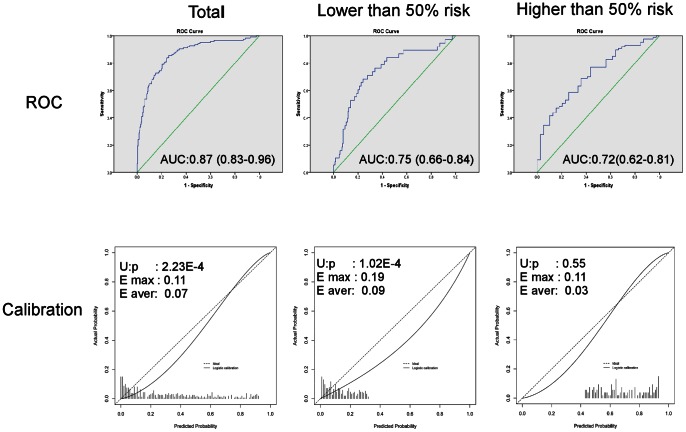
ROCs and calibrations of the total and 50% cut point subgroups of patients. The AUC of the total nodules was 0.87 (range, 0.83 to 0.90), but the calibration showed significant difference between the observed frequencies and predictive probabilities (p = 2.23*10^−4^). Based on the calibration plot, we used 50% as the threshold to divide the nodules into low risk and high-risk groups. In the low-risk group, the AUC was 0.75, and the calibration p-value was 1.02*10^−4^. But in the high-risk group, the AUC and calibration p-value were 0.72 and 0.55 respectively, which showed a good performance of the nomogram.

The AUC for the total nodules was 0.87 (range, 0.83 to 0.90), but the calibration p-value was 2.23*10^−4^. This result indicates that this model had good discrimination but cannot be well calibrated, indicating that the predictive probabilities were not concordant with the observed frequencies. Based on the calibration plot, we observed that the high-risk group (≥50%) appeared to have better calibration than the low-risk group (<50%) (Figure 1). Therefore, we used 50% as the threshold to identify the high-risk nodules. The AUC of the low-risk and high-risk groups were 0.75 and 0.72, respectively. The discriminations for the two subgroups were as good as that for the whole group. However, the calibrations of the two subgroups were tremendously different from each other. The calibration p-value of the low-risk and high-risk groups were 1.02*10^−4^ and 0.55, respectively (Table 3). The model only showed good performance for the high-risk group (≥50%).

**Table 3:** Summary of the ROCs and calibrations of the total and 50% cutoff point subgroups of thyroid nodules.

	Total	Lowerthan 50%	Higherthan 50%
No. of nodules	409	283	126
Malignant No. of nodules	125	38	87
Discrimination			
AUC of ROC	0.87	0.75	0.72
95%CI	0.83–0.90	0.66–0.84	0.62–0.81
Calibration			
p-value	2.23E–4	1.02E–4	0.55$
E max	0.11	0.19	0.11
E aver	0.07	0.09	0.03

AUC, area under the receiver operating characteristic curve; CI: coefficient interval; E: the difference between the predicted and calibrated probabilities; E max: maximal error; E aver: average error;

$p-value of higher than 50%: p>0.05 indicated that there is no difference between the predicted and calibrated probabilities, and it’s well calibrated.

## Discussion

With the widespread use of thyroid ultrasonography, the apparent incidence of thyroid nodules is increasing [17]. Most of these nodules are benign and do not require surgery [18]. However, the possibility of malignancy is greater if risk factors exist, such as a high TSH value [19], [20], hypoechogenicity, irregular margins, microcalcifications, increased nodular flow, a family history of carcinoma, radiation exposure and others [6], [21]. We used univariate and multivariate analyses to identify risk factors for malignancy (Table 2). The results were consistent with those of some authors [6], [21], [22], [23] but were inconsistent with the results of other authors [19], [20], [24]. Therefore, there is still controversy about whether these risk factors can be used as predictors of malignancy, and further investigation is needed.

Nomograms are statistical tools that can be used to determine diagnoses, predict prognoses or perform other clinical calculations by assessing the individual risks of patients. Several nomograms have been developed for the diagnosis of thyroid nodules [7], [12], [13], [14], [15]. Most of these models use the results of the thyroid FNAB as an important parameter. FNAB results can definitely improve the performance of thyroid nomograms. However, compared with the proportion in Western countries, the proportion of patients who undergo an FNAB in our hospital is much lower. Instead, ultrasonography has been employed as a screening examination to confirm the presence of thyroid nodules and to assess their malignancy. Because of its facility and relative high accuracy in cancer diagnosis, many surgeons perform diagnostic surgery based on ultrasonography. A nomogram can help the surgeon to select the patients who need FNAB for further investigation, and such assessments are mainly based on the clinical features of the thyroid nodules. This can help us to decrease the percentage of diagnostic surgery. Recently, Nixon [14] published such a nomogram for predicting the pathological diagnosis of patients with thyroid nodules without the FNAB. Nixon’s nomogram is based on eight clinical and thyroid ultrasonography characteristics, including age at diagnosis, TSH level, tumor shape, echo texture, calcification, margins, vascularity and tumor size. This nomogram has been validated internally, but predictive tools that were developed using a specific population may or may not be applicable to other patient cohorts [25]. Therefore, extensive external validations were needed to evaluate the usefulness of Nixon’s nomogram. This study aimed to externally validate this nomogram for use in the Chinese population. We identified patients using the same enrollment criteria used for the development of the nomogram. The results showed that the AUC was 0.87 (95% CI = 0.83–0.90), but the calibration p-value was less than 10^−4^ (p<10^−4^). We attempted to identify the main reason why the model could not be calibrated for all nodules, and we found that the difference in the distributions of the patients between our population and Nixon’s population contributed to this result. The percentage of malignant nodules in our study was 30.6% (125/409), and the percentage of benign nodules was 69.4% (284/409). However, the percentages in the population enrolled in the study introducing Nixon’s nomogram were 72% and 28%, respectively. Thus, the percentage of malignant nodules in our cohort was too low and the percentage of benign nodules was too high relative to the percentages in Nixon’s cohort. This difference might affect the correlation between the actual probabilities and the predicted probabilities. FNAB is an accurate method to differentiate benign from malignant thyroid nodules [26]. The use of FNAB can decrease the surgical rate by at least 25% and can increase the percentage of patients with malignant tumors who undergo surgery to more than 30% [27], [28]. Hence, the calibration could be improved if thyroid FNAB was used more frequently in Chinese patients because this technique can eliminate most of the unnecessary surgical procedures performed to remove benign thyroid nodules.

In a subsequent analysis, we found that the performance was better in the high-risk group (predictive probabilities ≥50%) because of the good discrimination (AUC = 0.72, 95% CI = 0.62–0.81) and calibration (p = 0.55). The low-risk group also had a good discrimination (AUC = 0.75, 95% CI = 0.66–0.84) but could not be calibrated (p = 10^−4^). Nixon’s nomogram performed better for the high-risk group than for the low-risk group in our population. We identified two reasons that contributed to these results. First, the percentage of malignant nodules in the high-risk group was 69% (86/126). This percentage is similar to that in the population used to construct Nixon’s nomogram. Therefore, the distributions of the two cohorts were similar. This similarity can explain the better calibration for the high-risk group to some extent. In the low-risk group, the percentage of malignant nodules was 13% (38/284), which was very different from that in the population used to construct Nixon’s nomogram. This difference might influence the calibration of Nixon’s nomogram for the low-risk group, as discussed above. Second, the American Thyroid Association guidelines recommend that thyroid ultrasonography be performed in all patients with palpable or nonpalpable nodular thyroid disease [29], [30]. The thyroid ultrasound characteristics of the thyroid nodules are the most important parameters in Nixon’s nomogram. Many previous studies have revealed that the sensitivity (percentage of malignant nodules diagnosed by thyroid ultrasonography as pathologically malignant nodules) of thyroid ultrasonography ranges from 75% to 97% and the specificity (percentage of benign nodules diagnosed by thyroid ultrasonography as pathologically benign) ranges from 43% to 74% [21], [31], [32], [33], [34], [35], [36], [37]. Although the sensitivity was higher than the specificity in those studies, a few articles have reported higher specificity than sensitivity [24], [38], [39]. A high sensitivity indicates that the diagnostic accuracy for malignant nodules is higher than that for benign nodules. In our study, we chose a 50% risk value as the cutoff point. Most of the nodules in the high-risk group were malignant, and most of those in the low-risk group were benign. Therefore, thyroid ultrasound-based diagnosis was more accurate in the high-risk group than in the low-risk group. Improving the accuracy of thyroid ultrasound-based diagnosis may enhance the performance of Nixon’s nomogram because of the high weight of the ultrasound results. These two reasons might explain why this nomogram could be calibrated for the high-risk group but could not be calibrated for the low-risk group.

In conclusion, we are the first to report the external validation of Nixon’s nomogram for the prediction of the pathological diagnosis of thyroid nodules in a Chinese population. The results demonstrate that Nixon’s nomogram is a valuable predictive model for Chinese cohorts. It has good performance for the high-risk group and may be more suitable for these patients in China.
